# Physical fitness and anthropometric normative values among Colombian-Indian schoolchildren

**DOI:** 10.1186/s12889-016-3652-2

**Published:** 2016-09-13

**Authors:** Jeison Alexander Ramos-Sepúlveda, Robinson Ramírez-Vélez, Jorge Enrique Correa-Bautista, Mikel Izquierdo, Antonio García-Hermoso

**Affiliations:** 1 Grupo de Investigación en Pedagogía, Licenciatura en Ciencias del Deporte y la Educación Física, Institución Universitaria Antonio Jose Camacho, Cali, Colombia; 2 Vicerrectoria de Investigaciones, Maestría en Ciencias y Tecnologías del Deporte y la Actividad Física, Universidad Manuela Beltrán, Bogotá, D.C Colombia; 3 Centro de Estudios en Medición de la Actividad Física (CEMA), Escuela de Medicina y Ciencias de la Salud, Universidad del Rosario, Bogotá, D.C Colombia; 4 Department of Health Sciences, Public University of Navarra, Pamplona, Spain; 5 Laboratorio de Ciencias de la Actividad Física, el Deporte y la Salud, Universidad de Santiago de Chile, USACH, Santiago, Chile; 6 Universidad San Sebastián, Santiago, Chile

**Keywords:** Physical fitness, Body composition, Obesity, Adolescent, Reference standards

## Abstract

**Background:**

Substantial evidence indicates that children’s physical fitness levels are markers of their lifestyles and their cardio-metabolic health profile and are predictors of the future risk of chronic diseases such as obesity, cardiometabolic disease, skeletal health and mental health. However, fitness reference values for ethnic children and adolescents have not been published in a Latin-American population. Therefore, the aim of the study was to provide sex- and age-specific physical fitness and anthropometric reference standards among Colombian-Indian schoolchildren.

**Methods:**

A sample of 576 participants (319 boys and 257 girls) aged 10 to 17 years old was assessed using the FUPRECOL test battery. Four components of physical fitness were measured: 1) morphological component: height, weight, body mass index (BMI), waist circumference (WC), triceps skinfold, subscapular skinfold, and body fat (%); 2) musculoskeletal component: handgrip and standing long jump test; 3) motor component: speed/agility test (4 × 10 m shuttle run); and 4) cardiorespiratory component: course-navette 20 m, shuttle run test and estimation of maximal oxygen consumption by VO_2_max indirect. Centile smoothed curves for the 3^rd^, 10^th^, 25^th^, 50^th^, 75^th^, 90^th^ and 97^th^ percentiles were calculated using Cole’s LMS method.

**Results:**

Our results show that weight, height and BMI in each age group were higher in boys than in girls. In each groups, age showed a significant effect for BMI and WC. Boys showed better than girls in cardiorespiratory fitness, lower- and upper-limb strength and speed/agility and girls performed better in low back flexibility.

**Conclusion:**

Our results provide for the first time sex- and age-specific physical fitness and anthropometric reference values for Colombian Nasa Indian children and adolescents aged 10–17.9 years.

## Background

Physical fitness is a multi-dimensional construct that includes skills and health related components, of which cardiorespiratory fitness (CRF) and muscular fitness in particular are powerful determinants of health in youth [1]. Substantial evidence indicates that children’s physical fitness levels are markers of their lifestyles and their cardio-metabolic health profile and are predictors of the future risk of chronic diseases [2–6], such for obesity, cardiovascular disease, skeletal health and mental health [1]. Physical fitness is also inversely associated with metabolic risk [7, 8] and is a valuable part of health monitoring in children [1, 8] and adults [9].

The inclusion of physical fitness within health surveillance systems is therefore clearly justifiable, and schools may be an ideal setting for the monitoring of youth fitness. Various health-related fitness test batteries are used to assess young people internationally, including the FUPRECOL battery [9], FITNESSGRAM battery [10], the President’s Challenge: Health Fitness [11], the EUROFIT [12], the CPAFLA [13], and the AFEA battery [14]. Recently, the Assessing Levels of Physical Activity (ALPHA) health-related fitness test battery was created as part of the framework of the EU- funded project, the HELENA Study “Healthy Lifestyle in Europe by Nutrition in Adolescence”, to be used in public health monitoring in a comparable way within the European Union [15]. The fitness tests included in the FUPRECOL health-related fitness battery assess the main components of fitness that have a relationship with health such as (a) morphological and body composition, (b) musculoskeletal fitness, (c) motor fitness, and (d) cardiorespiratory fitness.

However, race and ethnicity are also thought to be important determinants of health [16]. Both are terms used to categorize populations on the basis of shared characteristics. Race has traditionally been used to categorize populations on the basis of shared biological characteristics such as genes, skin color, and other observable features [17]. Ethnicity is used to categorize on the basis of cultural characteristics such as shared language, ancestry, religious traditions, dietary preferences, and history. Although ethnic groups can share a range of phenotypic characteristics because of their shared ancestry, the term is typically used to highlight cultural and social characteristics instead of biological ones [8, 11].

Indigenous populations have disparities in health along with disparities in modifiable risk factors, including a low participation in physical activity [8]. For instance, inequalities in nutrition may be related to race/ethnicity inequalities and household characteristics [18]. Although the relationship between race/ethnicity and obesity in children is inconsistent in developed countries, there appears to be a strong positive association between the two in developing countries [19]. The obesity-race/ethnicity association could vary by sex, age, and country [20]. Middle-income countries such as Colombia are experiencing rapid urbanization and integration into global markets, which leads to changes in diet and physical activity and, with these changes, large effects on body composition [9, 19]. These changes in body composition are contributing to a global increase in the prevalence of noncommunicable diseases. Data on the physical fitness of Indigenous youth are scarce; therefore the inclusion of fitness within health surveillance systems is clearly justifiable and may be an ideal setting for the monitoring of youth fitness in indigenous population. It is important to document the current state of physical fitness in Colombia, particularly among the most vulnerable groups including low income, rural populations, indigenous households, women and children.

Therefore, the aim of the study was to provide sex- and age-specific physical fitness and anthropometric reference standards among Colombian-Indian schoolchildren.

## Methods

### Participants and study design

Indigenous populations have disparities in health along with disparities in modifiable risk factors, including low participation in physical activity [8]. The present cross-sectional study was conducted to provide the distribution of health-related physical fitness across indigenous individuals. Data were collected in the Nasa community, located in the Toribío district, which is a town and municipality in Cauca Department, South Colombia. Nasa is considered an indigenous area, with approximately 90 % of the 186,178 inhabitants living in the surrounding rural areas. Data from the National Institute of Statistics in Colombia) indicate that the estimated prevalence of poverty is substantially higher in the Nasa indigenous (93 %) [21].

### Subjects

A sample of 576 (319 boys and 257 girls) healthy, Nasa Indian children and adolescents (10–17.9 years old) participated in the study. All the participants of the selected indigenous households were invited to participate in the study. The participation rate was greater than 95 %. A convenience sample of volunteers was included in groups by sex and age with 1-year increments (a total of 8 groups). The sample size was estimated at 15 to 30 participants per group. A power analysis showed that this sample size was sufficient to estimate the proportion of subjects with an aerobic capacity indicative of future cardiovascular risk (7 to 11 %) with a precision of 11.4 % and a power of 80 % by data from the FUPRECOL study [9]. The recruitment period lasted from June 2014 to January 2015.

#### Ethics approval and consent to participate

A comprehensive verbal description of the nature and purpose of the study and of the experimental risks was given to the participants and their parents/guardians. Parents/guardians were informed about the study via the school administration and could opt out if they disagreed with their schoolchildren participation. Participation in the study was fully voluntary and anonymous, with no explicit incentives provided for participation. Participants and their legal representatives received information sheets and all provided written informed consent and approval to take part in the study. The study was approved by the Review Committee for Research Human Subjects at the University of Manuela Beltrán (Resolution UMB N° 02-1902-2014). The protocol was in accordance with the latest revision of the Declaration of Helsinki.

### Procedures

#### Morphological component

Weight was measured to the nearest 0.1 kg. Height was measured to the nearest 0.1 cm. Waist circumference (WC) was measured by horizontally placing an inelastic tape measure midway between the lowest rib margin and the iliac crest to the nearest 0.1 cm. The instruments were calibrated to ensure acceptable accuracy. Body mass index (BMI) was calculated as body weight in kilograms divided by height squared in meters. The participants were categorized according to the BMI international cut-off values as normal weight, overweight, and obese [22]. During the anthropometric measurements, the students wore light clothing and were barefoot. Skinfold thicknesses (triceps and subscapular) were measured at the left side of the body to the nearest 0.1 mm using a Holtain skinfold caliper. The triceps thickness was measured at halfway between the acromion process and the olecranon process; subscapular thickness was measured approximately 20 mm below the tip of the scapula, at an angle of 45° to the lateral side of the body. Body fat (%) was also calculated using the formulas described by Slaughter et al. [23]. The predicted % body fat cut-off values for obesity used in this work by age and sex was 25–30 % in boys and 30–53 % in girls [24].

#### Musculoskeletal component

##### Explosive strength, standing broad jump (cm)

The participants stood behind the starting line and were instructed to push off vigorously and jump as far as possible. The participants had to land with their feet together and remain upright. The test was repeated twice, and the best score was retained. The farther of the two scores was recorded to the nearest 0.1 cm as the distance between toes at take-off and heels at landing, or whichever body part landed nearest to the take-off.

##### Handgrip strength (kg)

Handgrip strength was measured using a standard adjustable handle analogue handgrip dynamometer T-18 TKK SMEDLY III® (Takei Scientific Instruments Co., Ltd, Niigata, Japan). Handgrip strength was measured with the subject in a standing position with their shoulders adducted and neutrally rotated and their arms parallel but not in contact with the body. The participants were asked to squeeze the handle maximally for 3–5 s, but no verbal encouragement was given during the test. Two trials were performed on each side, alternately, with a rest period of at least 1 min between trials of the same hand. Thus, the reference values of handgrip strength presented here combine the results of left and right-handed subjects, without consideration of hand dominance.

##### Motor component: speed/agility test (speed of movement, agility and coordination assessment)

Two parallel lines were drawn on the floor 10 m apart. The adolescents ran as fast as possible from the starting line to the other line and returned to the starting line, crossing each line with both feet every time. This was performed twice, covering a distance of 40 m (4 × 10 m). Every time the adolescents crossed any of the lines, he/she would pick up (the first time) or exchange (second and third times) a sponge that had earlier been placed behind the lines. The stopwatch was stopped when the adolescents crossed the end line with one foot. The time taken to complete the test was recorded to the nearest tenth of a second. A slip-proof floor, four cones, a stopwatch and three sponges were used to perform the test.

#### Cardiorespiratory component

##### Cardiorespiratory fitness, 20-m shuttle run (ml•kg•min^−1^)

The participants ran in a straight line between two lines 20 m apart, while keeping pace with pre-recorded audio signals. The initial speed was 8.5 km/h and increased by 0.5 km/h per minute. The test was finished when the participant failed to reach the end lines keeping pace with the audio signals on two consecutive occasions or when the subject stopped because of fatigue. The results were recorded to the nearest stage (minute) completed. To estimate VO_2_max using the 20-m shuttle run, the equation developed by Leger et al. [25] was used: [VO_2max_ = 31.025 + 3.238 *V-3.248 *A + 0.1536 *V *A]. Here, “V” accounts for velocity (in km/h^−1^) of the last completed stage and “A” accounts for the subject’s age (in years) [25, 26].

#### Sexual maturation

Sexual maturation was classified on the basis of Tanner staging (self-reported pubertal status) as: prepubescent, pubescent, and postpubescent [27]. Each volunteer entered an isolated room, where, using a set of images exemplifying the various stages of sexual maturation, they categorized the development of their own genitalia (for boys), breasts (for girls), armpits (for boys) and pubic hair (for both genders). The reproducibility of our data reached 85 %. With regard to the communication processes and explanations of tests, direct and simple oral language was used. Additionally, the evaluators provided visual models and examples before performing the test, when necessary. The participants did not receive specific training on these tests previously.

All fit measurements in a subsample of (*n* = 124) boys and (*n* = 105) girls [mean weight = 46.2 ± 12.4 kg, mean height = 1.50 ± 0.1 m, mean BMI = 19. 9 ± 3.1, mean age = 12.8 ± 2.4 years] were repeated by undergoing the tests again 1 week later. The same inter-trial period has been used earlier in similar reliability studies conducted with healthy young individuals [28]. In all the tests, we found almost excellent test-retest reliability [waist circumference (ICC = 0.983), BMI (ICC = 0.973), triceps skinfold (ICC = 0.864), subscapular skinfold (ICC = 0.859), handgrip (ICC = 0.984), standing long jump test (ICC = 0.921), set and reach (ICC = 0.899), and *course-navette* shuttle run test (ICC = 0.967)], with the exception of the 4 × 10 m shuttle run, which obtained a moderate agreement (ICC = 0.685).

### Statistical analyses

All variables were checked for normality of distribution before analysis using histograms and Q-Q plots. None required transformation. The participants were divided into 8 age groups: 10 to 10.9, 11 to 11.9, 12 to 12.9, 13 to 13.9, 14 to 14.9, 15 to 15.9, 16 to 16.9 and 17 to 17.9 years. The age- and sex-specific values were reported as the mean ± the standard deviation (SD). We analyzed sex-group differences in the fitness variables by a one-way analysis of variance. To provide percentile values for the sample, we analyzed waist circumference, BMI, triceps skinfold, subscapular skinfold, handgrip, standing long jump test, 4 × 10 m shuttle run and course-navette shuttle run test data by maximum penalized likelihood using the LMS statistical method for boys and girls separately [29]. We derived smoothed centile charts using the LMS method. This estimates the measurement percentiles in terms of three age-specific cubic spline curves: the L curve (Box–Cox power to remove skewness), the M curve (median) and the S curve (coefficient of variation). The appropriate number of degrees of freedom was selected on the basis of the deviance, Q-tests and worm plots, following the suggestions of Royston & Wright [30]. The 3^rd^, 10^th^, 25^th^, 50^th^, 75^th^, 90^th^ and 97^th^ smoothing percentiles were chosen as the age- and gender-specific reference values. For the construction of the percentile curves, the data were imported into the LmsChartMaker software (V. 2.3; by Tim Cole and Huiqi Pan), and the L, M and S curves were estimated [31]. Except for the LMS method calculations, we used SPSS V. 21 software for Windows (SPSS, Chicago, IL, USA), and the significance level was set at 5 %.

## Results

### Descriptive characteristics

The characteristics for the four components of the FUPRECOL health-related fitness test according to the sex and age of the study sample are shown in Table 1. The mean values were as follows: age of 14.3 ± 2.2 years, weight of 46.1 ± 10.6 kg, height of 148.2 ± 11.6 m, BMI of 20.7 ± 2.7 kg/m^2^, waist circumference of 70.0 ± 7.3 cm, subscapular skinfold of 10.3 ± 3.7 mm, triceps skinfold of 12.5 ± 6.1 mm and body fat of 21.8 ± 5.5 %. The prevalence of overweight and obesity were significantly higher in girls (*p* = 0.001). Girls had a significantly higher % body fat and a significantly higher WC (*p* = 0.001). Handgrip strength, standing broad jump and VO_2_max were significantly different between sexes, and boys had significantly higher scores in the musculoskeletal component (*p* = 0.001), the cardiorespiratory component (*p* = 0.001) and the motor component (*p* = 0.001).

**Table 1:** Means and SD for Physical Fitness and Anthropometric Normative Values among Colombian-Indian Schoolchildren

	Total (*n* = 576)	Boys (*n* = 319)	Girls (*n* = 257)	*p* value
Morphologic component				
Age (years)	14.3 ± 2.2	14.4 ± 2.2	14.1 ± 2.2	0.198
Weight (kg)	46.1 ± 10.6	46.3 ± 11.3	45.8 ± 9.7	0.634
Height (m)	148.2 ± 11.6	150.4 ± 13.1	145.4 ± 8.8	0.000
Body mass index (kg/m^2^)	20.7 ± 2.7	20.1 ± 2.2	21.5 ± 3.1	0.001
Weight status *n* (%)				
Normal weight	494 (85.8)	192 (74.7)	302 (94.7)	0.001
Overweight	77 (13.4 )	62 (24.1)	15 (4.7)	0.001
Obese	5 (0.9)	3 (1.2)	2 (0.6)	0.001
Waist circumference (cm)	70.0 ± 7.3	69.1 ± 6.6	71.1 ± 8.0	0.001
Subscapular skinfold (mm)	10.3 ± 3.7	8.3 ± 2.6	12.8 ± 3.3	0.001
Triceps skinfold (mm)	12.5 ± 6.1	9.2 ± 3.2	16.6 ± 6.5	0.001
Body fat (%)	21.8 ± 5.5	19.8 ± 4.5	24.2 ± 5.7	0.001
Adiposity excess *n* (%)	113 (19.6)	33 (12.8)	90 (28.2)	0.001
Tanner stage *n* (%)				
Prepubescent	176 (30.6)	92 (35.7)	106 (33.2)	0.309
Pubescent	199 (34.6)	88 (34.3)	110 (34.5)	0.458
Postpubescent	200 (34.8)	77 (30.0)	107 (33.4)	0.674
Musculoskeletal component				
Handgrip (kg)	20.4 ± 7.7	23.2 ± 8.4	17.0 ± 4.8	0.001
Standing broad jump (cm)	151.6 ± 31.7	169.9 ± 28.3	128.9 ± 18.0	0.001
Motor component				
4 × 10 m shuttle run (s)	11.9 ± 1.2	11.3 ± 0.9	12.7 ± 1.0	0.001
Cardiorespiratory component				
20-m shuttle run (stage)	7.2 ± 2.5	8.6 ± 2.2	5.5 ± 1.8	0.001
VO_2_max (ml•kg•min^−1^)	47.7 ± 6.7	51.5 ± 4.7	43.1 ± 5.9	0.001

Mean ± standard deviation, except weight status and adiposity excess (%). Differences between boys and girls calculated using one-way analysis of variance and weight status future cardiovascular risk (X^2^ test)

### Normative values

**Fig. 1:**
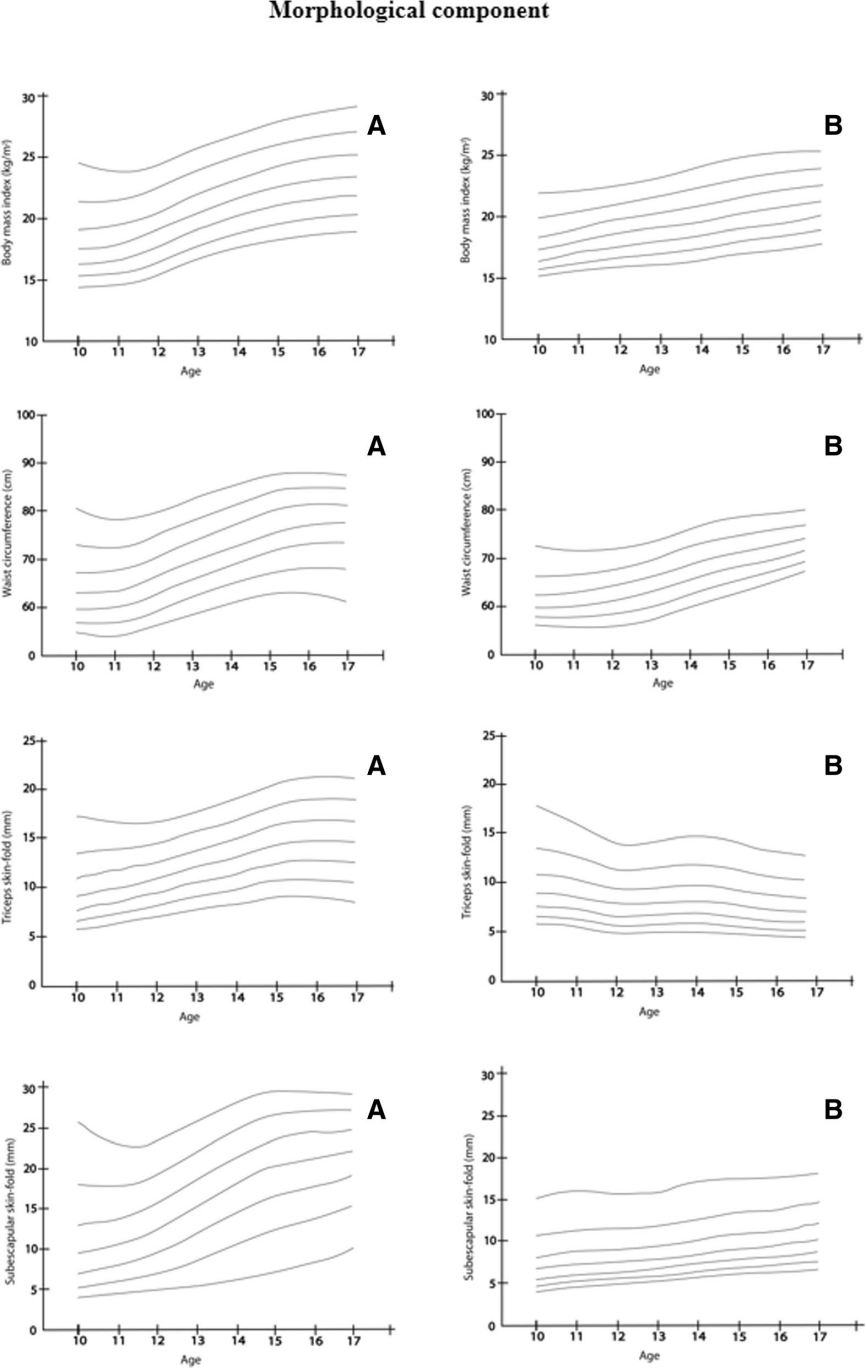
Smoothed (LMS method) centile curves (from the bottom to the top: P_3_, P_10_, P_25_, P_50_, P_75_, P_90_, P_97_) among Nasa Indigenous: morphological component. **a** boys and **b** girls 10–17.9 years of age

**Fig. 2:**
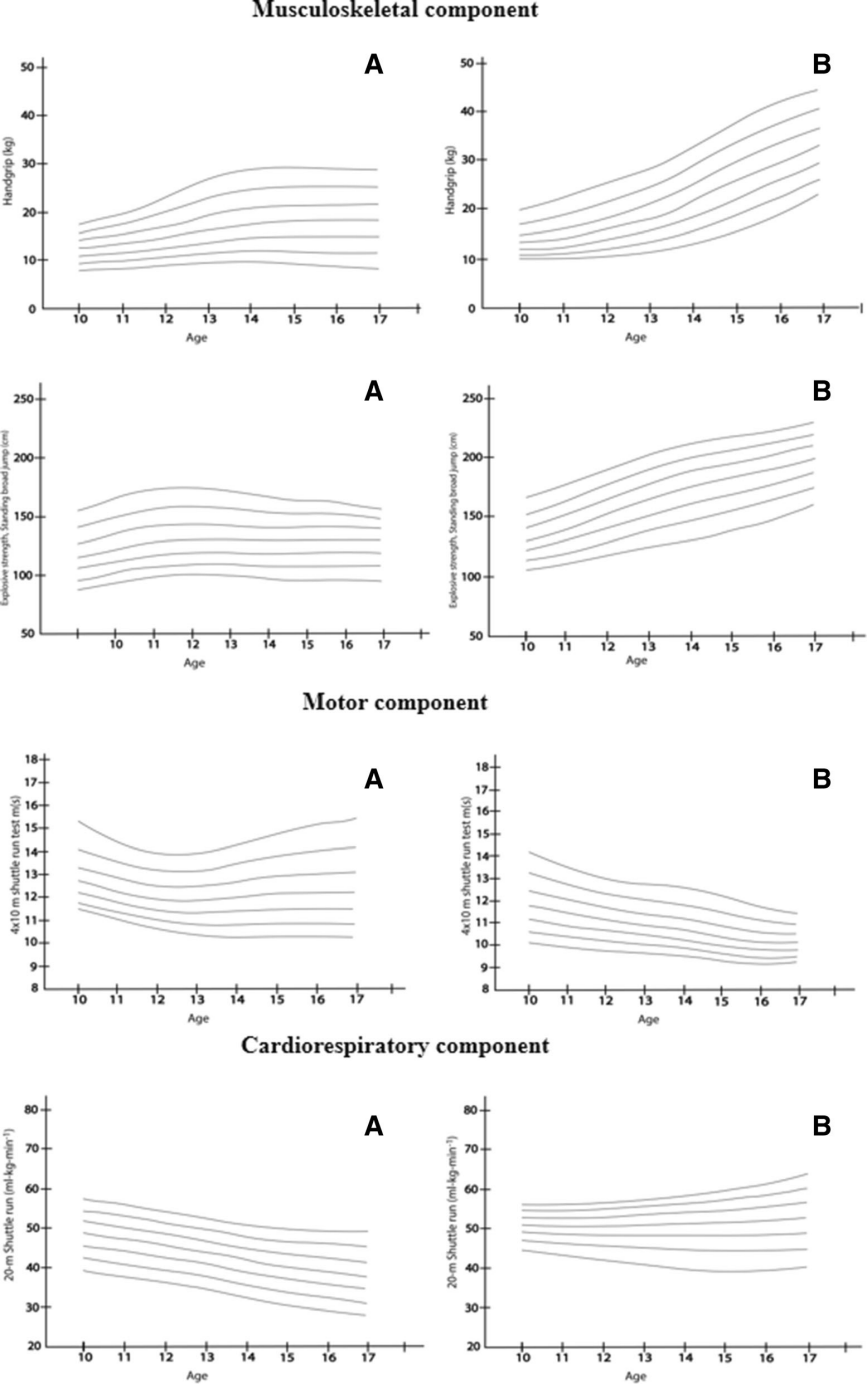
Smoothed (LMS method) centile curves (from the bottom to the top: P_3_, P_10_, P_25_, P_50_, P_75_, P_90_, P_97_) among Nasa Indigenous: Physical fitness component. **a** boys and **b** girls 10–17.9 years of age

Tables 2 and 3 show the normative values for waist circumference, BMI, triceps skinfold, subscapular skinfold, handgrip strength, standing long jump test, 4 × 10 m shuttle run and the *course-navette* shuttle run test in the Nasa Indian Community, classified according to sex and age and expressed in percentiles from 3 to 97. In boys, the BMI and WC 50^th^ percentiles ranged from 16.8 to 23.5 kg/m^2^ and 62.3 to 74.2 cm, respectively. In girls, the BMI and WC 50^th^ percentiles ranged from 16.1 to 20.1 kg/m^2^ and 64.0 to 76.5 cm, respectively (Fig. 1). Skinfolds in each age group were significantly higher in girls than in boys (Fig. 1). Boys performed better than girls in terms of the musculoskeletal component (handgrip strength, explosive lower body strength, and standing broad jump values) (Fig. 2). The mean 4 × 10 m shuttle-run values show a decreasing trend with age and sex, as shown in Table 3. The VO_2_max 50^th^ percentile (10 and 17 years) ranged from 51.2 to 52.8 ml•kg•min^−1^ in boys and from 37.2 to 49.1 ml•kg•min^−1^ in girls. Nevertheless, values show a decreasing trend with age in girls (Fig. 2).

**Table 2:** Percentile values summary statistics among Colombian-Indian Schoolchildren: Anthropometric outcomes

Age (boys)	*N*	Mean	SD	P_3_	P_10_	P_25_	P_50_	P_75_	P_90_	P_97_	Age (girls)	*N*	Mean	SD	P_3_	P_10_	P_25_	P_50_	P_75_	P_90_	P_97_
BMI (kg/m^2^)																					
10 to 10.9	27	19.8	3.4	15.9	15.9	16.8	18.6	22.8	25.8	26.1	10 to 10.9	24	17.5	1.9	15.3	15.3	16.1	16.6	18.8	20.6	20.6
11 to 11.9	31	18.6	1.5	15.1	16.8	17.6	18.6	19.9	20.6	20.8	11 to 11.9	23	18.6	1.5	16.2	16.6	17.7	18.5	19.3	21.4	21.5
12 to 12.9	58	17.9	1.7	14.6	15.3	17.0	17.8	18.9	19.9	22.1	12 to 12.9	31	18.8	1.8	15.9	17.0	17.6	18.5	19.5	21.0	24.8
13 to 13.9	32	20.9	2.3	17.1	18.1	18.9	20.7	22.1	23.7	26.2	13 to 13.9	39	19.4	1.7	15.8	17.5	18.0	19.5	20.7	21.3	23.8
14 to 14.9	38	21.2	2.1	17.6	18.8	19.7	21.1	22.7	24.5	26.4	14 to 14.9	33	19.7	2.0	15.9	16.9	18.9	19.7	20.2	21.9	26.0
15 to 15.9	49	22.8	2.2	19.3	19.7	21.0	22.8	25.0	25.7	26.8	15 to 15.9	32	20.2	1.4	17.2	18.6	19.2	20.2	21.4	21.7	22.7
16 to 16.9	32	23.6	2.5	18.4	19.7	21.9	23.6	25.6	26.9	27.3	16 to 16.9	33	21.3	2.6	18.3	18.7	19.4	20.8	22.8	24.0	31.2
17 to 17.9	52	24.0	2.7	19.6	20.5	21.7	23.5	25.7	27.4	30.0	17 to 17.9	42	21.6	1.9	18.1	18.7	20.1	21.4	23.1	23.8	25.8
WC (cm)																					
10 to 10.9	27	63.7	4.7	57.0	58.0	60.0	62.3	69.3	70.7	71.0	10 to 10.9	24	66.8	7.6	59.3	59.4	60.0	64.5	76.3	77.1	77.3
11 to 11.9	31	64.8	6.8	56.4	58.0	61.0	63.8	66.1	71.5	87.5	11 to 11.9	23	63.3	5.0	54.0	55.4	60.2	64.0	66.5	68.2	77.3
12 to 12.9	58	64.9	4.1	53.0	59.5	63.3	65.3	67.0	69.7	71.3	12 to 12.9	31	62.5	5.2	54.0	54.3	57.3	64.0	67.0	69.0	69.3
13 to 13.9	32	68.3	6.0	55.7	60.9	64.2	68.2	72.0	75.4	83.7	13 to 13.9	39	70.2	6.0	58.1	63.0	66.0	70.3	73.0	78.0	83.9
14 to 14.9	38	69.9	4.4	62.1	64.0	66.1	70.0	72.5	76.5	79.2	14 to 14.9	33	69.9	6.1	61.0	63.7	64.5	67.0	75.3	79.1	82.0
15 to 15.9	49	73.0	5.7	64.0	67.0	69.3	72.1	75.9	77.6	97.0	15 to 15.9	32	74.8	6.3	61.3	66.3	72.0	75.3	78.2	83.8	88.3
16 to 16.9	32	74.3	4.7	63.8	68.6	71.0	74.2	77.0	80.7	85.1	16 to 16.9	33	77.0	5.9	66.0	67.8	73.0	77.0	82.8	84.9	87.0
17 to 17.9	52	63.7	4.7	57.0	58.0	60.0	62.3	69.3	70.7	71.0	17 to 17.9	42	76.4	6.8	63.1	65.8	70.9	76.5	82.5	84.0	86.4
SS (mm)																					
10 to 10.9	27	6.3	2.0	4.0	4.0	5.0	6.0	8.0	10.0	10.0	10 to 10.9	24	13.0	7.5	3.0	4.2	7.0	10.0	19.0	24.2	30.0
11 to 11.9	31	8.5	2.6	4.3	5.5	6.5	8.0	11.3	12.3	13.0	11 to 11.9	23	11.3	3.6	6.0	7.0	9.0	11.0	13.0	16.9	21.0
12 to 12.9	58	8.2	3.8	5.0	5.3	6.2	7.2	9.0	11.0	23.6	12 to 12.9	31	9.8	3.0	7.0	7.0	7.3	9.3	12.0	14.8	17.0
13 to 13.9	32	8.0	2.2	5.0	5.1	6.5	7.7	9.0	11.0	15.0	13 to 13.9	39	15.0	5.5	3.1	7.0	12.0	15.0	20.0	21.3	25.2
14 to 14.9	38	8.7	3.1	5.0	6.0	7.0	8.0	9.3	11.0	20.2	14 to 14.9	33	16.2	5.3	8.0	9.4	12.2	15.0	20.0	25.2	27.0
15 to 15.9	49	9.7	3.1	6.0	7.0	8.0	9.0	10.5	14.0	19.2	15 to 15.9	32	19.0	5.3	4.0	12.6	17.3	18.3	21.2	27.4	29.0
16 to 16.9	32	9.8	2.8	7.0	7.0	7.3	9.0	12.0	13.7	18.0	16 to 16.9	33	21.1	5.5	10.0	11.6	15.7	22.0	25.5	27.6	28.0
17 to 17.9	52	10.6	2.7	7.0	7.0	9.0	10.2	12.0	14.7	16.8	17 to 17.9	42	21.3	5.0	7.1	14.5	18.0	22.3	25.0	26.7	29.0
TS (mm)																					
10 to 10.9	27	8.5	1.9	6.0	6.0	7.0	8.3	9.0	12.0	12.0	10 to 10.9	24	10.4	3.9	4.0	4.6	7.0	10.3	12.0	16.6	17.0
11 to 11.9	31	9.9	2.9	6.0	6.7	8.0	9.0	12.0	14.7	15.3	11 to 11.9	23	10.7	3.0	7.0	7.1	8.3	10.0	13.0	15.3	18.0
12 to 12.9	58	9.1	2.9	4.8	6.9	7.2	9.0	10.0	12.0	20.0	12 to 12.9	31	9.6	1.5	7.0	7.4	8.3	9.3	11.0	11.9	12.0
13 to 13.9	32	7.5	2.1	4.3	5.0	6.0	7.0	8.8	10.2	15.0	13 to 13.9	39	12.3	2.5	8.0	9.0	10.3	12.0	13.3	16.0	18.2
14 to 14.9	38	8.9	2.7	5.0	5.9	7.8	8.3	10.0	12.1	17.2	14 to 14.9	33	12.8	2.2	8.0	10.0	11.0	13.0	14.7	15.6	16.3
15 to 15.9	49	8.3	1.9	5.0	6.0	7.0	8.0	9.0	10.3	12.7	15 to 15.9	32	14.1	3.7	5.0	10.1	12.0	14.0	15.0	19.8	24.0
16 to 16.9	32	8.1	3.4	5.0	5.0	6.0	7.0	10.0	11.0	23.0	16 to 16.9	33	14.6	2.3	11.0	11.4	13.0	14.0	16.0	18.0	20.0
17 to 17.9	52	7.5	1.8	4.6	5.3	6.0	7.0	9.0	10.0	10.8	17 to 17.9	42	14.7	3.5	5.5	11.0	13.3	14.3	16.3	19.0	23.2

*M* mean, *SD* standard deviation, *P* percentile, *BMI* body mass index, *WC* waist circumference

**Table 3:** Percentile values summary statistics among Colombian-Indian Schoolchildren: Physical Fitness outcomes

Age (boys)	*N*	Mean	SD	P_3_	P_10_	P_25_	P_50_	P_75_	P_90_	P_97_	Age (girls)	*N*	Mean	SD	P_3_	P_10_	P_25_	P_50_	P_75_	P_90_	P_97_
CRF (ml•kg•min^−1^)																					
10 to 10.9	27	52.4	2.7	48.4	48.4	50.4	52.8	55.3	55.9	55.9	10 to 10.9	24	48.6	4.8	43.2	43.3	45.6	48.0	51.4	57.5	60.8
11 to 11.9	31	50.2	3.4	43.9	45.2	46.9	51.6	53.9	54.0	54.1	11 to 11.9	23	49.1	4.5	41.3	43.1	44.5	49.1	53.9	54.4	56.3
12 to 12.9	58	50.7	3.3	43.1	46.0	48.0	51.2	53.0	53.5	58.0	12 to 12.9	31	46.3	5.3	35.4	39.8	41.2	47.4	51.0	53.0	53.7
13 to 13.9	32	51.2	3.8	37.8	45.2	51.2	51.9	53.4	54.5	57.3	13 to 13.9	39	45.1	3.8	36.4	40.8	41.8	45.9	46.7	51.4	51.8
14 to 14.9	38	50.7	4.3	40.1	44.9	47.5	51.6	53.2	55.9	58.6	14 to 14.9	33	43.0	4.3	31.9	37.5	39.5	42.9	45.5	49.4	50.5
15 to 15.9	49	51.7	5.0	37.9	45.5	49.0	52.2	54.7	57.4	60.0	15 to 15.9	32	40.2	4.8	31.4	32.6	37.2	40.5	43.4	46.5	49.2
16 to 16.9	32	51.3	4.8	39.3	44.9	47.4	52.6	55.7	56.2	58.3	16 to 16.9	33	41.4	4.8	27.6	34.3	38.4	42.1	45.0	46.4	50.0
17 to 17.9	52	52.8	5.5	40.2	45.5	49.1	52.2	57.6	59.6	62.1	17 to 17.9	42	38.5	5.7	29.4	32.7	34.0	37.2	42.8	45.9	54.7
Standing broad jump (cm)																					
10 to 10.9	27	131.3	11.4	119.0	119.0	120.3	132.0	146.0	146.3	146.3	10 to 10.9	24	111.9	13.1	88.3	91.2	104.0	110.2	126.0	130.2	130.4
11 to 11.9	31	136.5	14.3	110.4	118.2	127.1	134.8	149.2	157.6	165.0	11 to 11.9	23	124.0	17.7	92.0	105.5	110.6	116.3	138.0	151.7	160.3
12 to 12.9	58	143.2	19.3	108.4	122.0	129.8	142.3	151.3	165.7	198.7	12 to 12.9	31	131.6	20.6	91.6	108.3	117.2	128.8	150.0	166.0	166.3
13 to 13.9	32	161.4	19.7	128.8	130.2	144.3	165.0	175.2	188.5	191.3	13 to 13.9	39	131.2	19.5	102.1	106.6	114.7	126.3	146.2	157.0	171.9
14 to 14.9	38	165.9	19.8	128.1	144.4	150.2	162.7	181.1	194.1	201.4	14 to 14.9	33	136.7	16.6	114.0	119.4	122.4	134.0	152.2	158.8	171.9
15 to 15.9	49	180.0	18.6	136.0	152.3	168.0	183.0	195.5	202.3	211.0	15 to 15.9	32	121.7	15.1	80.0	99.4	113.7	119.7	133.2	139.0	150.0
16 to 16.9	32	186.4	19.3	137.0	156.5	176.3	189.0	200.0	204.4	223.0	16 to 16.9	33	131.0	15.2	106.0	108.4	121.0	133.0	139.5	151.8	165.8
17 to 17.9	52	194.8	17.7	157.0	170.0	180.5	198.0	207.5	216.4	224.0	17 to 17.9	42	130.7	17.0	97.2	106.7	118.0	132.0	142.6	157.3	163.7
Handgrip (kg)																					
10 to 10.9	27	13.5	0.8	12.2	12.2	12.5	13.9	13.9	13.9	13.9	10 to 10.9	24	13.5	2.6	6.4	8.8	12.7	13.9	14.2	17.3	17.5
11 to 11.9	31	14.6	2.6	9.7	11.4	13.4	13.9	16.9	18.9	20.4	11 to 11.9	23	13.9	2.1	10.9	11.1	12.0	13.9	14.5	17.6	18.7
12 to 12.9	58	15.9	3.8	10.9	11.5	13.6	14.7	17.6	20.7	26.1	12 to 12.9	31	13.7	2.6	7.5	9.5	12.7	13.9	15.2	16.5	19.1
13 to 13.9	32	18.0	4.3	10.9	12.7	15.1	17.2	20.6	25.4	27.7	13 to 13.9	39	16.6	5.0	10.3	11.1	13.0	15.3	19.8	24.7	28.4
14 to 14.9	38	19.2	4.0	10.8	13.6	16.4	19.3	22.5	23.8	26.7	14 to 14.9	33	17.7	4.0	9.5	12.0	15.4	17.7	20.4	22.8	25.4
15 to 15.9	49	24.0	4.8	14.5	17.8	20.5	23.5	27.6	30.6	34.7	15 to 15.9	32	19.8	5.4	5.6	13.2	16.3	19.9	24.0	26.9	30.1
16 to 16.9	32	28.6	6.7	14.0	19.2	24.3	27.4	34.1	36.1	46.4	16 to 16.9	33	17.5	4.8	10.1	12.0	13.7	17.0	20.7	23.9	32.2
17 to 17.9	52	32.7	4.9	21.3	25.7	29.0	32.9	36.4	38.5	42.7	17 to 17.9	42	19.4	5.3	8.2	11.7	16.3	19.3	22.5	26.8	30.1
4 × 10 m run (s)																					
10 to 10.9	27	12.6	1.6	15.2	15.2	14.2	12.3	11.2	10.9	10.9	10 to 10.9	24	13.6	1.1	16.4	15.7	14.2	13.3	12.8	12.5	12.3
11 to 11.9	31	12.2	0.7	14.5	13.1	12.7	12.1	11.7	11.4	11.2	11 to 11.9	23	13.2	0.8	15.0	14.6	13.7	13.2	12.5	12.3	12.0
12 to 12.9	58	11.8	0.7	13.4	12.6	12.3	11.7	11.3	10.8	10.7	12 to 12.9	31	12.6	0.7	15.0	13.2	12.9	12.6	12.3	11.6	11.3
13 to 13.9	32	11.6	0.7	13.1	12.8	12.0	11.5	10.9	10.7	10.5	13 to 13.9	39	12.3	0.7	14.0	13.0	12.8	12.5	11.7	11.4	11.2
14 to 14.9	38	11.5	0.6	12.9	12.5	11.8	11.5	10.8	10.7	10.5	14 to 14.9	33	12.3	1.1	14.9	14.2	12.9	12.2	11.6	10.9	10.2
15 to 15.9	49	11.2	0.8	13.3	12.3	11.5	11.0	10.6	10.4	10.0	15 to 15.9	32	12.9	0.8	14.8	13.8	13.5	13.0	12.4	11.7	11.4
16 to 16.9	32	10.8	0.7	13.0	12.0	11.4	10.5	10.4	10.2	9.8	16 to 16.9	33	12.8	1.4	16.9	14.2	13.3	12.7	11.7	11.1	10.7
17 to 17.9	52	10.7	0.7	12.9	11.5	10.9	10.6	10.2	10.1	9.4	17 to 17.9	42	12.6	1.1	16.1	13.8	13.2	12.6	11.8	11.3	10.9

*M* mean, *SD* standard deviation, *P* percentile, *CRF* cardiorespiratory fitness

Finally, comparisons between the 50^th^ percentile or mean values for physical fitness outcomes from our study and other international studies are presented in Table 4. We found that Colombian-Indian schoolchildren have higher values of CRF, standing broad jump and 4 × 10 m run except in handgrip test than children and adolescents from Colombia, UK and the European Union (EU).

**Table 4:** Reference values (50^th^ percentile or mean) for physical fitness from cited studies

Age (boys)	Present study *n* = 576	Colombia [72, 76] *n* = 7268	UK [77, 78] *n* = 7147	EU [46]^a^ *n* = 3428	Age (girls)	Present study *n* = 576	Colombia [72, 76] *n* = 7268	UK [77, 78] *n* = 7147	EU [46]^a^ *n* = 3428
CRF (ml•kg•min^−1^)^c^									
10 to 10.9	52.8	22.0	47.1^b^	51.6^b^	10 to 10.9	48.0	16.0	44.7^b^	46.8^b^
11 to 11.9	51.6	23.5	45.9^b^	51.1^b^	11 to 11.9	49.1	18.0	43.0^b^	47.5^b^
12 to 12.9	51.2	27.0	45.3^b^	51.9^b^	12 to 12.9	47.4	20.0	41.9^b^	46.6^b^
13 to 13.9	51.9	34.0	45.2^b^	50.0^b^	13 to 13.9	45.9	21.0	40.7^b^	44.4^b^
14 to 14.9	51.6	40.0	46.6^b^	50.1^b^	14 to 14.9	42.9	23.0	39.2^b^	41.6^b^
15 to 15.9	52.2	48.0	45.7^b^	50.2^b^	15 to 15.9	40.5	22.0	37.2^b^	41.1^b^
16 to 16.9	52.6	52.0	46.4^b^	49.9^b^	16 to 16.9	42.1	25.0	37.8^b^	39.5^b^
17 to 17.9	52.2	54.0	–	49.6^b^	17 to 17.9	37.2	25.0	–	38.6^b^
Standing broad jump (cm)									
10 to 10.9	132.0	118.0	138.0	–	10 to 10.9	110.2	102.0	126.0	–
11 to 11.9	134.8	123.0	143.0	–	11 to 11.9	116.3	107.0	133.0	–
12 to 12.9	142.3	126.0	149.0	–	12 to 12.9	128.8	110.0	140.0	–
13 to 13.9	165.0	139.0	156.0	159.0	13 to 13.9	126.3	114.0	145.0	140.0
14 to 14.9	162.7	148.0	166.0	176.0	14 to 14.9	134.0	115.0	150.0	144.0
15 to 15.9	183.0	158.0	178.0	189.0	15 to 15.9	119.7	115.0	154.0	145.0
16 to 16.9	189.0	163.0	189.0	199.0	16 to 16.9	133.0	117.0	156.0	147.0
17 to 17.9	198.0	165.0	–	208.0	17 to 17.9	132.0	120.0	–	150.0
Handgrip (kg)									
10 to 10.9	13.9	14.1	16.6	–	10 to 10.9	13.9	13.4	15.5	–
11 to 11.9	13.9	15.6	19.6	–	11 to 11.9	13.9	15.3	18.7	–
12 to 12.9	14.7	17.5	22.6	–	12 to 12.9	13.9	18.1	21.2	–
13 to 13.9	17.2	21.1	27.2	26.2	13 to 13.9	15.3	19.5	23.5	23.6
14 to 14.9	19.3	23.8	32.5	32.2	14 to 14.9	17.7	21.9	25.8	25.2
15 to 15.9	23.5	28.5	39.0	37.7	15 to 15.9	19.9	21.5	26.9	26.2
16 to 16.9	27.4	31.1	–	41.8	16 to 16.9	17.0	22.7	–	26.6
17 to 17.9	32.9	33.5	–	45.1	17 to 17.9	19.3	23.3	–	27.6
4 × 10 m run (s)									
10 to 10.9	12.3	13.8	–	–	10 to 10.9	13.3	15.0	–	–
11 to 11.9	12.1	13.8	–	–	11 to 11.9	13.2	14.5	–	–
12 to 12.9	11.7	13.4	–	–	12 to 12.9	12.6	14.4	–	–
13 to 13.9	11.5	13.1	–	12.0	13 to 13.9	12.5	14.5	–	12.8
14 to 14.9	11.5	12.9	–	11.7	14 to 14.9	12.2	14.3	–	12.7
15 to 15.9	11.0	12.5	–	11.2	15 to 15.9	13.0	14.3	–	12.7
16 to 16.9	10.5	12.3	–	10.9	16 to 16.9	12.7	14.5	–	12.6
17 to 17.9	10.6	11.9	–	10.9	17 to 17.9	12.6	14.3	–	12.6

*CRF* cardiorespiratory fitness. ^a^EU: from 10 European cities in Austria, Belgium, France, Germany, Greece (an inland city and an island city), Hungary, Italy, Spain and Sweden

^b^mean. ^c^VO_2peak_ (ml•kg^−1^•min^−1^) predicted using the Leger et al. equation [25]. – Not reported

## Discussion

The main objectives of this study were to provide sex- and age-specific physical fitness and anthropometric reference standards among Colombian-Indian schoolchildren. These results showed that the boys performed better than the girls in speed, lower- and upper-limb strength and cardiorespiratory fitness. However, this is the first published research study using the FUPRECOL test battery [9] in a sample of Colombian indigenous youths. The main strength of this study, and in terms of the normative values hereby provided, is the strict standardization of the fieldwork among the Indian community.

### Normative values

Anthropometric indicators are useful both at an individual and population level. At an individual level, anthropometric body indicators can be used to assess compromised health or nutrition wellbeing. At the population level, body composition can be used to assess the nutrition status within a country, region, community, or socioeconomic group, and to study both the determinants and consequences of malnutrition and/or other risk factors [32]. This form of monitoring is valuable both for the design and targeting of health and nutrition interventions, particularly among minority populations [33].

This study shows a prevalence of overweight, including obesity, of 14.3 % in boys and 25.3 % in girls according to BMI. The present results clearly show greater and more homogenous anthropometry body composition data in girls, except for BMI, in which the values in boys were slightly higher. Therefore, BMI cutoffs appear to be a good criterion for the screening of excess body fat in adolescents; however, an important percentage of subjects classified as overweight or obese did not really have excess adiposity [34].

In Colombia, the major part of the indigenous community living in high-poverty areas has been associated with a higher prevalence of overweight/obesity and hypertension, even after controlling for physical activity, BMI, and occupation [35]. Nevertheless, guidelines exist to identify, evaluate, and treat overweight/obese children [36], but there is insufficient evidence to recommend a specific treatment approach according to the race/ethnicity of the child. Although the implication of a given BMI is known to differ by race and ethnicity in adults, analogous data are lacking in children [17].

Expert panels have recommended measuring triceps and subscapular skinfold thicknesses as part of the in-depth medical assessment of children and adolescents; age- and gender-specific BMI cut-off values of the 95^th^ percentile or 30 (whichever was smaller) or age- and gender-specific BMI cut-off values of the 85^th^ percentile but <95^th^ percentile or equal to 30 (whichever was smaller) were suggested. In this study, girls had a significantly higher % body fat and a significantly higher WC (*p* = 0.001). Similar mean body fat values between age groups were observed in American [37] and Brazilian [38] girls, which showed an increase in the amount of body fat from the 25^th^ percentile to the 75^th^ percentile. Naturally, because of the action of sexual hormones, a progressive increase in body fat was observed in girls with maturation; however, their lower involvement in physical activities [39] and inadequate eating habits [40] may contribute to the increased body fat percentage levels as well. According to Mihalopoulos et al. [37], this increase in body fat could be related to physical and sexual development, which is in line with results obtained in other research.

### Physical fitness

In 2009, Ruiz et al. [41] systematically reviewed whether fitness in childhood was a predictor of cardiovascular disease risk factors, events and syndromes, quality of life and low back pain later in life. The present values may be useful in identifying adolescents at a higher risk for developing unfavorable health outcomes owing to their low fitness level. Recently, Ruiz, et al. [42] showed that the 5^th^ percentile to the 25^th^ percentile of levels of physical fitness was identified as the “pathological fitness level” or as a “warning sign”; thus, youth in or below the 25^th^ percentile should be examined to determine whether they have a cardiovascular risk factor. Reports have shown that low levels of self-reported physical activity or low cardiorespiratory fitness are associated with insulin resistance in other indigenous groups [43, 44]. However, few studies available in the literature have investigated physical fitness profiles with similar socio-cultural characteristics and from the same ethnic origin [45].

Previously in adolescent populations, Ortega et al. [46] first published European fitness reference values for 12.5–17.5-year-old youths from 10 cities (HELENA study) and reported sex- and age-specific physical fitness levels. Our results were not comparable to those from Ortega et al. [46] for adolescents aged 10–17.9 years. For example, the P_50_ of the standing long jump was 172.0 cm vs 120.5 cm for Colombian Nasa Indian and European boys, respectively, and 127.0 cm vs 110.7 cm for Nasa Indian and European girls, respectively. The P_50_ of handgrip was an average of 21.5 kg vs 11.8 kg for Nasa Indian and European boys, respectively, and 16.2 kg vs 10.8 kg for Nasa Indian and European girls, respectively. In addition, the P_50_ of the 20-m shuttle run (stage) was 8.0 vs 2.0 for Nasa Indian and European boys, respectively, and 6.0 vs 2.0 for Nasa Indian and European girls, respectively. Likewise, Tremblay et al. [47] reported normative data for aerobic fitness and muscular strength for Canadian 11–19-year-old youths; however, only the 50^th^ percentile was reported. For example, in the study of Tremblay et al. [47], the performance in the 20-m shuttle run (VO_2_max in ml•kg•min^−1^) at the 50^th^ percentile of 11–14-year-olds was 54.9 ml•kg•min^−1^ and 48.9 ml•kg•min^−1^ in boys and girls, respectively. In the present study, the performance in VO_2_max at the 50^th^ percentile of 11–17.9-year-olds was 51.9 ml•kg•min^−1^ and 43.3 ml•kg•min^−1^ for boys and girls, respectively. In addition, in the study of Tremblay et al. [47], the performance in handgrip strength at the 50^th^ percentile was 25 kg and 22 kg in boys and girls, respectively, which was calculated as the sum of the best right- and left-hand attempts. In our study, performance in the handgrip test at the 50^th^ percentile of 10–17.9-year-olds was 21.5 kg and 16.2 kg for boys and girls, respectively. In terms of speed/agility, the values for boys were similar to those of three studies (Spanish [48], European [46] and Norwegian [49]), whereas the girls’ performance was lower than that of the three analyzed studies.

Previous research indicates that such low fitness levels can linger on into adulthood in which low cardiorespiratory fitness or low muscular strength is associated with increased mortality risk [50, 51]. However, differences in the environment alone do not appear to tell the entire story, particularly in elucidating why certain populations and ethnic groups experience a disproportionately high prevalence of cardiovascular disease when they adopt a Western lifestyle. The classic example of this is the Pima Indians, who when living a traditional rural lifestyle in Mexico are lean, active and have a low diabetes prevalence, but when living in the US, are generally obese and have prevalence of diabetes mellitus and other noncommunicable diseases in the adult population of ~40 % [52]. This pattern is also evident in other indigenous populations throughout the Americas and Australasia [53].

Compared to Nasa Indian adolescents in another studies, our sample had a significantly better performance in the musculoskeletal (SBJ), cardiorespiratory and motor components. Therefore, explaining the differences between urban and rural adolescents remains speculative and studies on the topic from other countries are inconsistent. One possible explanation for the differences in physical fitness profiles among indigenous adolescents may be the differences in moderate to vigorous physical activity [54] or differences in body fat distribution [55]. Probably, children living in rural areas may have more opportunities for active play or active transportation, as well as a reduced access to technologies such as the internet, which contributes to those children meeting the recommendations for physical activity [56]. Also, urban youth have more barriers that can restrict the freedom of children living in these areas, including spaces for playing and practicing physical activities. Additionally, in the rural areas, the youth normally helped their parents in work activities, reducing the time available for sedentary leisure activities. Specifically, the community of Cauca is characterised by smallholders and family agriculture, which in theory allows for the greater inclusion of youth in the daily work activities of their parents.

Physical activity and fitness have been found to be independently associated with certain cardiovascular risk factors among children and adolescents [57]. Contrarily, in Latin-American, urban Ecuadorian adolescents had better physical fitness and blood lipid profiles than rural adolescents, independent of time spent sedentary [58]. In summary, the observed high prevalence and sex differences of unhealthy cardiorespiratory fitness, particularly in girls, may be explained by the behavioral and environmental risk factors to which the Nasa community is currently exposed, including diet, lifestyle, and smoking. The lower levels of physical fitness and the excess weight in girls may stem from excess caloric consumption, lower caloric expenditure (due to reduced physical activity), or both, which may produce a vicious cycle. However, other environmental and socio-economic correlates must be explored.

### Limitations and strengths

There are a few limitations of this study. Firstly, the cross-sectional nature we cannot discern the direction of the observed associations between physical fitness and future cardiovascular risk, which may indeed be reciprocal. Adolescents with healthy body composition and healthy physical fitness may be more likely to engage in physical activity, which may lead to healthier cardiorespiratory and muscular fitness and contributes to the prevention of obesity [52, 53]. Furthermore, the maintenance of muscle mass, as indicated by healthy muscle fitness, can contribute to higher resting metabolic rate and consequently have a preventive effect on fat mass accumulation [58]. Secondly, we did not measure important variables associated with blood lipids such as levels of physical activity, sex hormone levels, and familial health background. Third, the estimation of VO_2_max from the FITNESSGRAM standards of the 20 m-shuttle run is known to vary with the equation used. A previous study [59] has tested the degree of agreement between various equations used to estimate VO_2_max and the actual the VO_2_max. We were unable to confirm, for example, that the observed associations remain after control for physical activity or pubertal stage as others have shown due to lack of such data in this study. However, such limitations do not compromise the results obtained when validating these results. Finally, the small number of studies on the indigenous population did not allow us to make comparisons with the results of this work. The majority of the published fitness reference values are for children from high income countries in North America [60–62], Asia/Oceania and Europe, [63–68], whereas there is a scarcity of reference values for children using harmonized measures of fitness in Latin-America [69] and other low- and middle-income countries (LMICs) undergoing a nutrition transition, [70] making impossible to evaluate secular trends within these regions. Colombia is in the midst of a nutrition transition that is mirroring the changes occurring elsewhere in the world, such as an increase in the overweight/obese population and a general decrease in its chronically undernourished population [68]. Furthermore, despite their larger burden of chronic disease and the alarming increase in the prevalence of obesity in children in Colombia, [69] for example, LMICs are also substantially underrepresented in physical activity intervention research [71, 72]. Previously, in indigenous populations, we have reported that children living in rural areas and in geographic regions with lower economic and structural development generally have lower serum concentrations of micronutrients than in children from urban areas [73–76]. Likewise, in the dietary study we conducted in Villamor [77], only 13.4 % of the protein in the diets of children was derived from eggs and milk, with 40 % of protein coming from meat. Colombia is a country that is geographically, climatically and ethnically diverse. Clearly, these differences could affect food supply, dietary practices, and consequently micronutrient intakes. Indigenous and other ethnic groups of Colombia are very diverse, which leads to different dietary patterns and climates. Colombia’s rapid economic development and modernization has led to the “westernization” of the Colombian diet, characterized by an increase in the absolute number of calories, saturated fat, and fast food consumed, and a decrease in the consumption of legumes, fruit, and cereals [77]. These dietary patterns may partially explain our results, given the effect of diet on physical fitness in adolescents.

On the other hand, our decision to categorize physical fitness according to health predictive value instead of using continuous variables can be considered a strength of the study as it allowed for greater public health interpretability. Another potential strength of the study was the use of four health-related, valid, and reliable field tests recommended for Latin-American youth fitness assessment [78]. Finally, we provided an accurate description of the physical fitness and anthropometric characteristics of the Colombian indigenous youths and their age-related variations.

## Conclusion

In summary, indigenous boys showed better than girls in cardiorespiratory fitness, lower- and upper-limb strength and speed/agility and girls performed better in low back flexibility. These values may be useful in identifying adolescents being at higher risk for developing unfavourable health outcomes owing to their low fitness level (<10^th^ percentile). Also, these cut-offs are especially interesting in educational setting due to school could play a major role in helping to identify adolescents with low physical fitness. Simultaneously, this study allows for more accurate categorization, which considers a youth age and gender, and enables comparisons among normative values from other countries. In addition, they are important because monitoring health-related physical fitness early in life might contribute to substantial improvements in life expectancy and reduced risk of chronic diseases such as obesity, cardiometabolic disease, skeletal and mental health in the indigenous population.

## Abbreviations

ALPHA: Assessing Levels of Physical Activity
BMI: Body mass index
CRF: Cardio-respiratory fitness
EUROFIT: European Physical Fitness Test Battery
HELENA: Healthy Lifestyle in Europe by Nutrition in Adolescence Study
ICC: Intraclass correlation coefficient
LMICs: Low- and middle-income countries
LMS: L curve (Box–Cox power to remove skewness), M curve (median), S curve (coefficient of variation)
SD: Standard deviation
